# Electrode Surface Heating with Organic Films Improves CO_2_ Reduction Kinetics on Copper

**DOI:** 10.1021/acsenergylett.4c00204

**Published:** 2024-03-11

**Authors:** Nicholas B. Watkins, Yungchieh Lai, Zachary J. Schiffer, Virginia M. Canestraight, Harry A. Atwater, Theodor Agapie, Jonas C. Peters, John M. Gregoire

**Affiliations:** † Liquid Sunlight Alliance, California Institute of Technology, Pasadena, California 91125, United States; ‡ Division of Chemistry and Chemical Engineering, California Institute of Technology, Pasadena, California 91125, United States; § Division of Engineering and Applied Science, California Institute of Technology, Pasadena, California 91125, United States

## Abstract

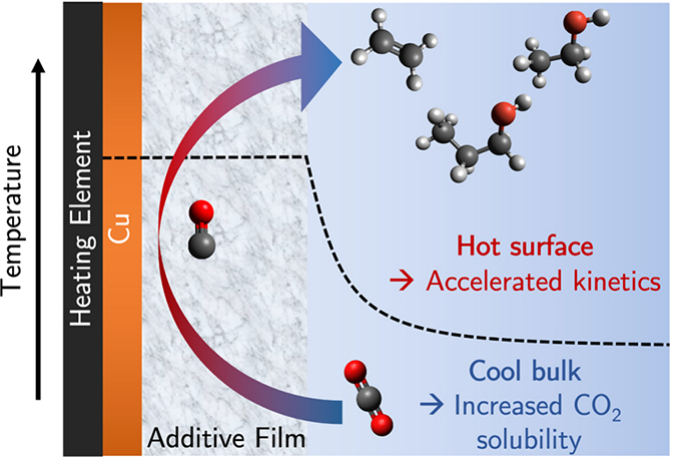

Management of the electrode surface temperature is an understudied aspect of (photo)electrode reactor design for complex reactions, such as CO_2_ reduction. In this work, we study the impact of local electrode heating on electrochemical reduction of CO_2_ reduction. Using the ferri/ferrocyanide open circuit voltage as a reporter of the effective reaction temperature, we reveal how the interplay of surface heating and convective cooling presents an opportunity for cooptimizing mass transport and thermal assistance of electrochemical reactions, where we focus on reduction of CO_2_ to carbon-coupled (C_2+_) products. The introduction of an organic coating on the electrode surface facilitates well-behaved electrode kinetics with near-ambient bulk electrolyte temperature. This approach helps to probe the fundamentals of thermal effects in electrochemical reactions, as demonstrated through Bayesian inference of Tafel kinetic parameters from a suite of high throughput experiments, which reveal a decrease in overpotential for C_2+_ products by 0.1 V on polycrystalline copper via 60 °C surface heating.

Decarbonization of the chemical industry is an important step toward halting the progress of anthropogenic climate change. Electrochemical reactions driven with solar power and other renewable energy sources to manufacture commodity chemicals, such as ammonia, ethylene, and hydrogen, have been recent targets to achieve this goal.^1^ While these commodity chemicals are currently being produced by well-established thermochemical processes, such as the Haber-Bosch process, each product has a clear alternative electrochemical synthetic pathway.^2^ Ammonia can be produced via nitrogen reduction (or Haber–Bosch with electrochemically produced hydrogen), ethylene can be produced via CO_2_ reduction (CO_2_R), and hydrogen can be produced via water reduction (Scheme 1).^3,4^ In addition, while the simplest operation is to drive these processes using grid-based renewable electricity alone, eventual electrolyzers can be integrated with solar-driven cells to afford photoelectrochemical (PEC) devices that directly harness the sun’s energy and enable distributed chemical manufacturing. While these processes historically have not been economically viable, the development of improved catalysts, membranes, photovoltaics, and government incentives drive forward their feasibility.^5^

**Scheme 1:**
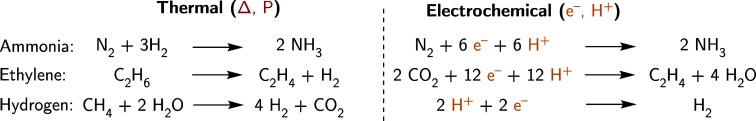
Comparison of the Thermal and Electrochemical Pathways for the Production of Ammonia, Ethylene, and Hydrogen In the electrochemical transformations, the reductive reaction listed is implicitly paired with an oxidative reaction such as oxygen evolution from water.

Thermocatalysis involves thermally activated traversal of a reaction barrier, which is well described by the Arrhenius expression for the rate constant *k* (eq 1).^6,7^ Here, *A* is a pre-exponential factor, *E*_*a*_ is the activation energy for the reaction, *R* is the universal gas constant, and *T* is the temperature of the reaction. Since lowering the activation energy is not always possible, methodologies for increasing reaction temperature are therefore desirable.
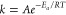
1The electrochemical analogue to eq 1 is the simplified Butler–Volmer expression for the kinetic current (*i*_*k*_) at high driving forces where the reverse reaction is negligible, often termed the Tafel equation:
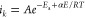
2In the Tafel equation, in addition to the same temperature-dependent exponential with an activation energy, there is also a linear, potential-dependent term in the exponential. More complex theories expand on Butler–Volmer by, for example, adding a quadratic potential term to the exponential, as is done with the Marcus theory. Here, α is the transfer coefficient, which is a function of the pre-equilibrium electron transfers and the rate-determining step, and *E* is the electrostatic potential with respect to a reference potential.^7,8^ We note that the pre-exponential factor *A* may also vary with temperature, which is not considered in the present work. In this case, the Tafel equation retains the qualitative form of the traditional Arrhenius expression, and elevated temperatures will increase the kinetic current.^9^

Since elevated temperature will improve reaction kinetics, the question remains how to efficiently heat the system. Industrial water splitting and CO_2_ reduction processes heat the entire electrolyzer to 40–60 °C and operate at current densities of or above 500 mA/cm^2^.^10,11^ It is of note that the limitation for these operating temperatures is typically the stability of the membrane and not of the catalyst.^12^ While uniform heating is beneficial for homogeneous reactions associated with many traditional thermochemical processes, electrochemistry is localized to the electrode surface; heating the bulk may therefore result in wasted energy. Additionally, resistive heating at industrially relevant current densities causes electrode surface temperature variation from the bulk by more than 10 °C.^13,14^ In photoelectrochemically driven systems, irradiative heating can cause local heating of the electrode surface by a similar margin.^15^ Given the sensitivity of electrochemistry to changes in temperature, these differences between set point and actual electrode temperature may have significant impacts on catalysis.

Bulk heating experiments in electrochemical CO_2_ reduction on copper have shown variable results. While all reports show increasing hydrogen and decreasing methane at elevated temperatures, ethylene promotion has varied between studies.^16−19^ We expect that this discrepancy may be due to variable convective mass transport between systems, which has been shown to have a significant effect on selectivity at 25 °C and would become especially important at elevated temperatures due to decreased CO_2_ solubility.^20−22^ There is evidence from the electrochemical sensor literature that enhanced reactivity can be achieved by using local heating.^23−25^ In the case of CO_2_ reduction, this would overcome the trade-off associated with decreasing bulk CO_2_ solubility.^20^ Recently, this concept has been applied to CO_2_R catalysis with both surface heating and cooling, achieving altered performance without significantly affecting the bulk temperature.^26,27^ In these works, Bi rotating disk electrodes (RDEs) increased their activity for formate by a factor of 1.7 upon raising surface temperatures to 65 °C and planar Cu electrodes boosted their methane selectivity to 80% by cooling the electrode to −4.4 °C (and applying pulsed electrolysis). In contrast to previous works, surface heating on copper showed no clear trend in ethylene or methane Faradaic efficiencies with respect to temperature, especially in the absence of supporting EDTA in the electrolyte, supporting the fact that hydrodynamics can significantly impact performance.^27^ In this work, we evaluate how mass transport and electrodeposited organic films affect the performance of heated electrodes for ferricyanide and CO_2_ reduction to C_2+_ products.

To establish a system with variable electrode temperature and hydrodynamics, we expanded the high throughput analytical electrochemistry (HT-ANEC) screening system to include a Peltier heating element that is electrically isolated and thermally coupled to a planar working electrode. To characterize the behavior of the cell with a heated working electrode and electrolyte flow, we invoked multiphysics modeling to establish the distribution of electrolyte flow rate and temperature throughout the working electrode chamber (Figure 1).^28^ The design of the cell varies slightly from our previous report on the effects of hydrodynamics on Tafel slopes to allow for a thermocouple to be placed inside the working compartment to monitor internal temperature.^22^ We measured internal and outlet temperatures at five temperature points with surface heating (SH) to evaluate the degree of global heating of the system. At a surface temperature of 60 °C, we experimentally measure an internal temperature of 36 ± 1.1 °C and an outlet temperature of 26.8 ± 0.1 °C, which supports our goal of mitigating bulk electrolyte heating. Our simulations further support this claim, with the average temperature in the cell showing Gaussian temperature distributions at temperatures far below the surface temperature (Figure S1 and Table S1).

**Figure 1:**
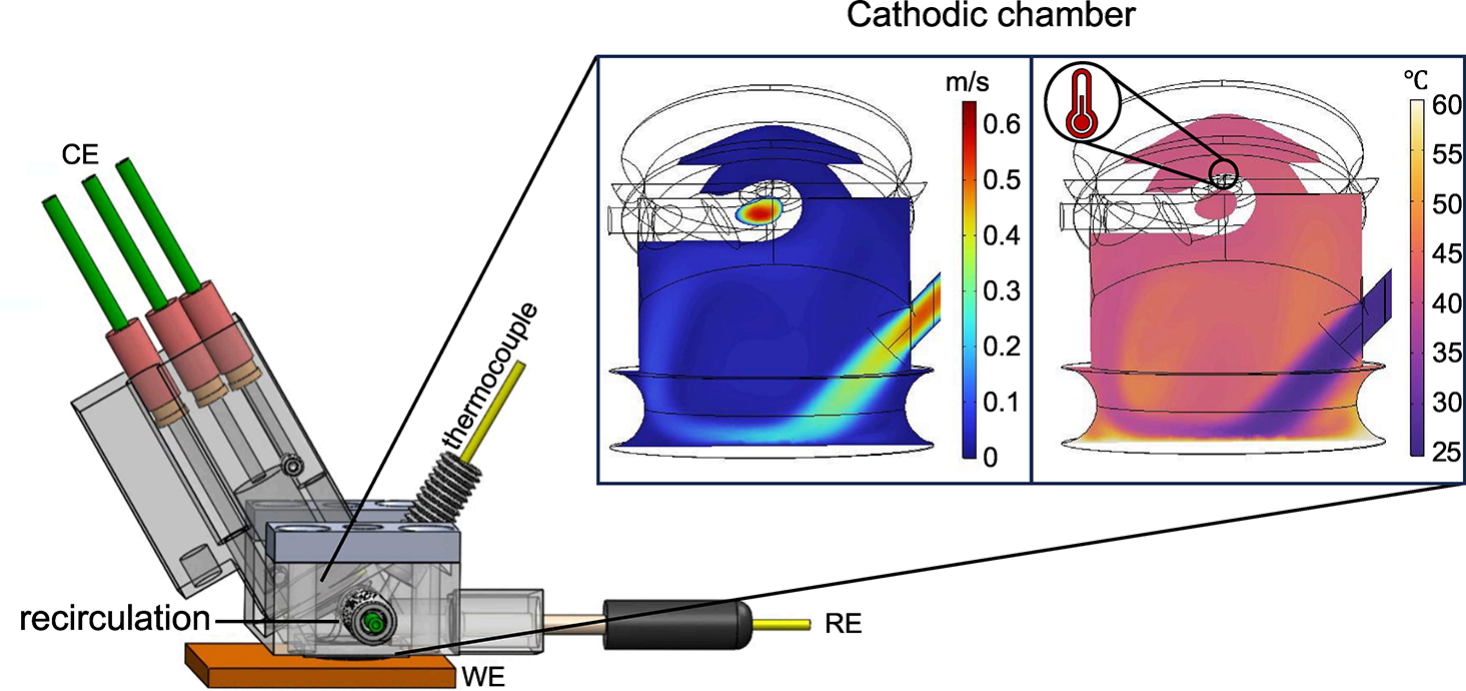
(A) Schematic of the high throughput analytical electrochemistry (HT-ANEC) screening system utilized in this report. The working electrode is placed on top of a Peltier heating element to accurately modulate surface temperature, and the internal temperature can be monitored using a thermocouple inserted in the top of the cell. In the inset are cross sectional images of the simulated velocity and temperature profiles within the cell given a flow rate of 150 μL s^–1^ and a surface temperature of 60 °C. In the thermal inset, we indicate the position of the thermocouple in the cathodic chamber.

To characterize the effective temperature of electrochemical reactions under the condition with a heated working electrode and ambient recirculating electrolyte, we measured the open circuit potential with an electrolyte containing equal concentrations of potassium ferri/ferrocyanide, whose temperature-dependent equilibrium potential is well established.^29^ We performed open circuit voltage (OCV) measurements at our standard flow rate of 150 μL s^–1^ as well as a reduced flow rate (Figure 2A). While the observed temperatures reflect the expectation that rapidly flowing ambient electrolyte lowers the effective reaction temperature with respect to the electrode temperature, these deviations are within ca. 5 °C (Figure 2B, Table S2, and Figure S2) and demonstrate our ability to systematically vary with reaction temperature via electrode heating. To further understand the differences between surface and bulk heating, we identified the mass transport limited current for each heating system by performing constant potential electrolyses at variable temperatures and using Fick’s second law to determine the average concentration boundary layer (δ_C_) thickness (Figure S3–5).^30^ Upon changing the temperature, we find that the δ_C_ decreases in thickness for both systems but marginally less with SH, which we expect is due to incomplete/inhomogeneous heating of the concentration boundary layer with SH (Figure S6). Partial heating is also consistent with the changes in cell resistance, since we observe slightly lower resistances with BH than SH. (Figure S7).

**Figure 2:**
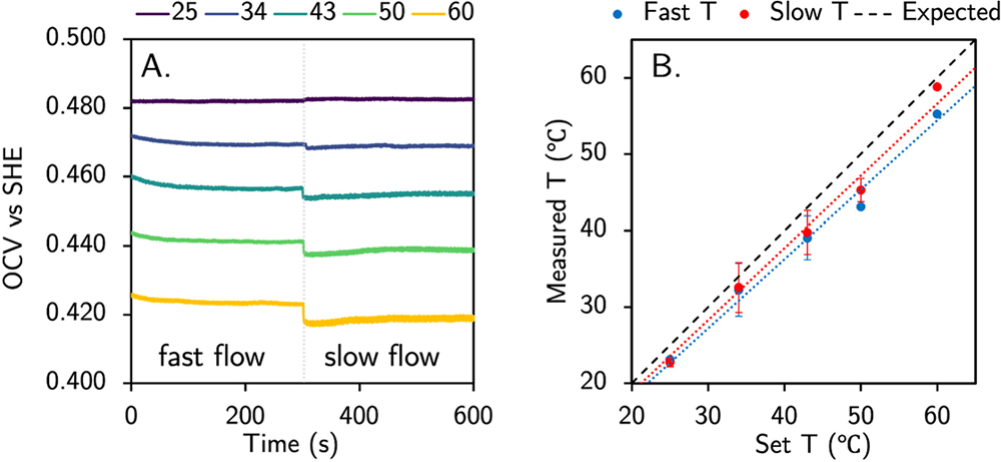
(A) OCV measurements at variable electrode temperatures over time changing from a fast electrolyte recirculation rate to a slower one at 300 s. (B) Comparison of measured temperature values for the two recirculation rates compared to the set temperatures. Error bars indicate the variance between the two measurements for each temperature. Electrochemistry was performed using a sputtered platinum film working electrode, a platinum wire counter electrode, and a leakless Ag/AgCl reference electrode, in 0.5 M KCl with 5 mM K_3_Fe(CN)_6_ and 5 mM K_4_Fe(CN)_6_.

Applying surface heating in electrocatalytic CO_2_R trials, we observe an increase in activity for both CO_2_R and HER, which is consistent with all previous reports (all FEs in Figure S8, Table S3).^16−19^ With respect to carbon-coupled products, we see a 2× increase in partial current density and up to 10% increase in Faradaic efficiency at −1.03 V vs RHE (Figure 3A).^16,19^ We observe no appreciable improvement in C_2+_ partial current density heating the surface from 43 to 60 °C, supporting the hypothesis from Koper et. al that other factors, such as structural changes, may be significant factors at these elevated temperatures.^19^ Unexpectedly, we did not observe a noticeable shift in onset potential for C_2+_ products. Since the shift in *J*_*CO2R*_ with respect to temperature is only slight, we expect that the more significant increase in *J*_*HER*_ at more positive potentials convolutes the system’s CO_2_R response to temperature, for example via competition for active sites (Figure S9). Temperatures above 80 °C were unable to be tested on bare copper due to the total current density exceeding the limitations of the HT-ANEC screening system.^28^

**Figure 3:**
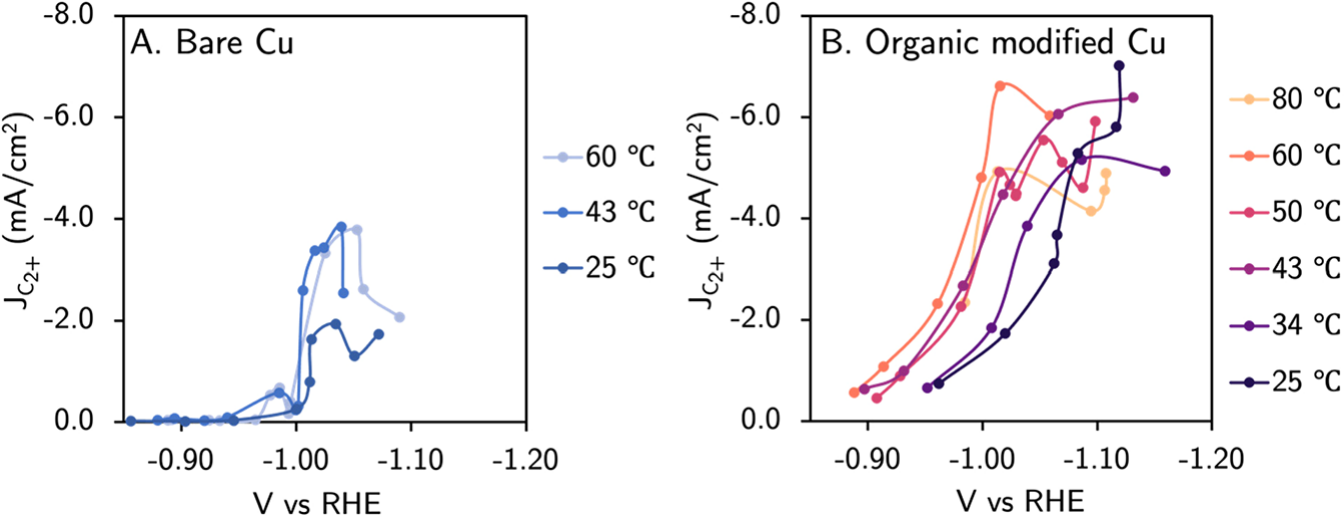
Electrochemical CO_2_ reduction performance (A) without and (B) with organic films in 0.1 M KHCO_3_. Each data point corresponds to an individual experiment. The organic film was deposited via a 10 min predeposition of 10 mM diphenyliodonium triflate at −1.2 V vs RHE in CO_2_-sparged 0.1 M KHCO_3_. 10 mM diphenyliodonium was present during electrolysis in the case of the additive film as well to heal minor delamination, as reported previously.^31^

In our previous work, we determined organic films improve CO_2_R performance toward multicarbon products by decreasing the availability of water while increasing the local concentration of CO.^22^ We hypothesized that the addition of an organic coating in this work would eliminate convoluting effects from competing hydrogen evolution and enable investigation of temperature-dependent CO_2_R. While previous investigations with organic coatings in this electrochemical cell were derived from N,*N*′-ethylenephenanthrolinium dibromide, herein we investigate films from the reductive electrodeposition of diphenyliodonium triflate due to their increased robustness (Scheme 2).^31^ Upon the incorporation of an organic film, we observe a boost in C_2+_ FE and a systematic increase in activity for CO_2_ reduction with the temperature. Notably, we observe a clearer positive trend in *J*_*CO2R*_ with respect to temperature with additives than without (Figure S10). Since we observe little change in concentration boundary layer thickness with respect to surface temperature (Figure S6), this result supports that the CO_2_ concentration, or chemical potential, is unchanged. Consequently, we infer that the observed temperature-dependent partial current densities reflect changes to the activation energy barriers in traditional reaction rate models, such as eqs 1 and 2.^7^ Commensurate with this hypothesis, we observe a positive shift in onset potential for carbon-coupled product formation (Figure 3B; all FEs in Figure S11 and Table S3). The highest activity for C_2+_ products was observed at −1.02 V vs RHE and SH = 60 °C, where we obtained a FE_C2+_ of 44% and a partial current density of 6.61 mA cm^–2^. At ambient temperature, an additional 0.1 V of overpotential is needed to obtain comparable C_2+_ activity, highlighting how temperature-based improvements to electrode kinetics enable operation at lower overpotentials. We observe a change in slope for the response in current with respect to potential with and without molecular additives, which is consistent with our previous report on how transport affects the electrode kinetics observed on polycrystalline copper.^22^ The systematic improvement to C_2+_ activity is observed up to 60 °C, above which we suspect that the loss in enhancement may be from delamination of the organic coating or the restructuring of copper.^19^ The data up to this temperature provide the opportunity to model the temperature-dependent Tafel equation (eq 2) while remaining cognizant of noise in the data, which may arise from, for example, inhomogeneities in mass transport across the electrode surface. In the present work, we are ultimately not concerned with the uncertainty in the performance at a given electrochemical condition but rather the uncertainty in the parameters of a model that describes the performance across all electrochemical conditions. We thus turn to Bayesian methods to infer the uncertainty in model parameters under consideration of the scatter in the experimental data. We present an anecdotal characterization of single-condition reproducibility in Figure S12.

**Scheme 2:**
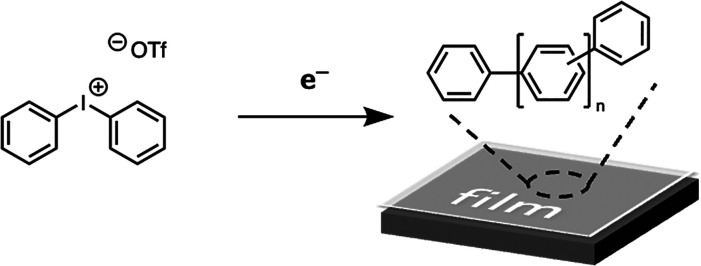
Under Reductive Bias, Diphenyliodonium Polymerizes on the Electrode Surface to Form a Robust Polyaromatic Coating That Is Electronically Insulating but Permeable to Reactants and Solvent^31^

While the Tafel expression is analogous to a traditional Arrhenius rate constant expression, calculating the activation energy for an electrochemical reaction is nontrivial because any temperature-dependent analysis (such as plotting log_10_(*i*_*k*_) vs 1/*T*) will result in the calculation of a convolution of activation energy, transfer coefficient, and applied potential. Specifically, the slope on a log_10_(*i*_*k*_) vs 1/*T* plot is not the activation energy as it is with a thermochemical reaction but instead is the quantity (−*E*_*a*_ + *αE*). Thus, to calculate the apparent activation energy of an electrochemical reaction, a comprehensive analysis of a range of potentials and temperatures is necessary, which is seldom done due to limitations in sufficient data collection for rigorous parameter estimation procedures. This consideration guided our design of combinatorial experimentation to characterize the transition in onset potential across temperatures and fit the resulting data to a temperature-dependent Tafel model coupled with a mass-transfer limiting current (Figure S13).^8,22^ Using the data collected with organic-coated Cu at a range of temperatures and potentials, we established a Bayesian model for the posterior distributions for all model parameters (see the SI for discussion and derivation). The result is an apparent activation energy of 1.0 ± 0.2 eV for the reduction of CO_2_ to C_2+_ products (Figure 4a), which differs from previously reported values (ca. 0.5 eV) that were established with different methodology. Herein we explicitly model *E*_*a*_, while previous analyses report the value of the expression (−*E*_*a*_ + *αE*).^18^ We note that carbon-coupled products are aggregated in this analysis due to their presumed common rate-determining step and corresponding activation barrier. In addition to the apparent activation energy, we concomitantly model the rate of change of the onset potential with a changing temperature (Figure 4B) and the rate of change of the current with a changing temperature (Figure 4C). These derivatives reveal that with increasing temperature, the overpotential at fixed C_2+_ current is lowered at a rate of ca. 2 mV K^–1^. At fixed overpotential, the C_2+_ current increases exponentially at a rate of 0.02 dec K^–1^. Overall, the estimation of these values and derivatives for CO_2_ reduction is only possible with the breadth of data achievable with HT-ANEC as well as comprehensive analysis of the complete data set with an accurate model for the current as a function of temperature and voltage. Furthermore, we find that organic modification was essential to enable the calculation of these fundamental parameters. While this study was limited to CO_2_R on organic modified Cu, the integration of combinatorial experimentation and Bayesian analysis can be used to determine activation barriers for a myriad of electrochemical reactions.

**Figure 4:**
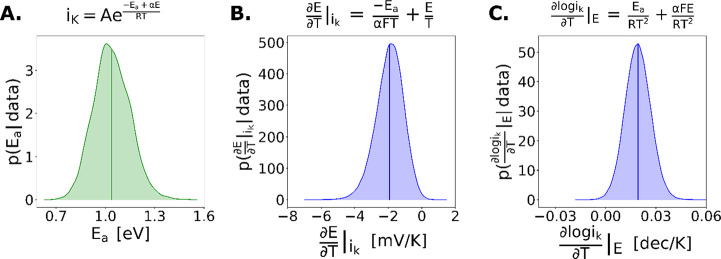
Probability distributions of the (A) activation energy, *E*_a_, for CO_2_ reduction with molecular films using surface heating, (B) observed change in applied potential with respect to temperature given a fixed kinetic current, and (C) observed change in kinetic current with respect to temperature given a fixed applied potential.

The use of surface heating and organic coatings herein demonstrates a methodology for identifying the apparent activation energy of an electrochemical transformation while mitigating the influence of bulk mass transport. Combining this technique with automated experimentation, we demonstrate that the ensemble of partial current densities acquired at various potentials and temperatures can be modeled by the temperature-dependent Tafel equation. By invoking Bayesian methods, the uncertainty in model parameters can also be inferred, which in the present work yields an apparent activation energy for C_2+_ products of 1.0 ± 0.2 eV, which is deconvoluted from the transfer coefficient and applied potential. With this methodology, we enable future systematic catalyst screening for lower C_2+_ barriers and subsequent system design around low *E*_*a*_ catalysts to achieve high activity and selectivity for carbon-coupled products at reduced overpotentials.

## Supporting Information Available

Detailed methods, modeling information, additional electrochemical data, additional fitting data, and a data table containing the temperature-dependent electrochemical and product distribution data used for parameter fitting (PDF)
