# Supplementary material for: Electrode Surface Heating with Organic Films Improves CO2 Reduction Kinetics on Copper
Source: ACS Energy Lett. 2024 Mar 11;9(4):1440–5. doi: 10.1021/acsenergylett.4c00204 (PMC11019637; doi:10.1021/acsenergylett.4c00204)
Supplement: Supplementary file 1 — nz4c00204_si_001.pdf [file nz4c00204_si_001.pdf]

Electrode Surface Heating with Organic Films Improves CO_2 Reduction Kinetics on Copper

Nicholas B. Watkins,[†a,b] Yungchieh Lai,[†a,c] Zachary J. Schiffer,[a,c,‡] Virginia M. Canestraight,[a,b] Harry A. Atwater,[a,c] Theodor Agapie,[a,b,*] Jonas C. Peters,[a,b,*] John M. Gregoire[a,c,*]
[a] Liquid Sunlight Alliance, California Institute of Technology, Pasadena, CA, 91125, USA.
[b] Division of Chemistry and Chemical Engineering, California Institute of Technology, Pasadena, CA, 91125, USA
[c] Division of Engineering and Applied Science, California Institute of Technology, Pasadena, CA, 91125, USA

*Email: agapie@caltech.edu, jpeters@caltech.edu, gregoire@caltech.edu

† These authors contributed equally to this work.

‡ Present address: Applied Physics, Harvard John A. Paulson School of Engineering, Harvard University, Cambridge, MA 02138

Supplemental Information

Materials and methods……2
Synthesis of diphenyliodonium triflate……2
High-throughput electrochemical testing……2
COMSOL modeling information……3
Parameter estimation for CO_2 reduction……3
Figure S1: Internal temperature comparison……4
Table S1: Internal temperature comparison……4
Figure S2: Temperature-dependent ferri/ferrocyanide OCV calibration……5
Table S2: Electrode microenvironment temperatures using OCV……5
Figure S3: Cyclic voltammograms of ferricyanide at variable surface temperatures……6
Figure S4: Chronoamperometry of ferricyanide at variable surface temperatures……7
Figure S5: Concentration boundary layer thickness at variable flow rates at 25 ℃……8
Figure S6: Concentration boundary layer thickness at variable surface temperatures……8
Figure S7: Temperature vs resistance plot of surface and bulk heated systems……9
Figure S8: Variable temperature and potential CO_2R FE on bare polycrystalline Cu……10
Figure S9: CO_2R partial current density on bare polycrystalline Cu……10
Figure S10: Variable temperature and potential CO_2R FE on additive-modified polycrystalline Cu……11
Figure S11: CO_2R partial current density on additive-modified polycrystalline Cu……11
Figure S12: Reproducibility of ethylene formation at select condition……12
Figure S13: Fitting of experimental data to Butler-Volmer kinetics (Equation 2 in main text)……13
Table S3: All raw data……14-15
References……16

Materials and Methods

All solvents and reagents were obtained from commercial sources and used as received, unless stated otherwise. Cu foils (99.999% Cu, 25 mm × 25 mm × 0.5 mm), potassium carbonate (99.995%), potassium ferricyanide (≥99%), potassium ferrocyanide hydrate (99.95%) were purchased from Sigma Aldrich. Carbon rods (99.999% C) were purchased from Strem Chemicals. Platinum foil (99.99% Pt, 25 mm × 25 mm × 0.05 mm), was purchased from Alfa Aesar. Natural abundance CO_2 (Research grade) was purchased from Airgas. Water was purified by a Nanopure Analytical Ultrapure Water System (Thermo Scientific) or a Milli-Q Advantage A10 Water Purification System (Millipore) with specific resistance of 18.2 MΩ·cm at 25 °C.

Prior to each use, copper foil was mechanically polished to a mirror-like finish using nanodiamond suspension (first 3 µm then 0.1 µm, Buehler) followed by rinsing in water and drying under a stream of nitrogen gas. The copper foil was then electropolished using a method similar to the one employed by Kuhl *et al.*: In a 85% phosphoric acid bath, +2.1 V versus a carbon rod counter electrode was applied to the Cu foil for 5 minutes and the foil was subsequently washed with copious amounts of ultra-pure water and dried under a stream of nitrogen gas. Prior to each use, platinum foil was washed with water and flame-annealed using a butane torch for 10 s. CO_2-saturated potassium bicarbonate electrolyte ($KHCO_3$, 0.1 M) was prepared by sparging an aqueous solution of potassium carbonate (K_2CO_3, 0.05 M) with CO_2 for at least 1 h prior to electrolysis.

Synthesis of Diphenyliodonium Triflate

The synthesis of diphenyliodonium triflate was performed as reported.[1] The as-synthesized compound was characterized by 1H NMR in d6-DMSO (400 MHz) δ(ppm): 8.26 (d, J = 7.0 Hz, 1H), 7.68 (t, J = 7.5 Hz, 1H), 7.54 (t, J = 7.8 Hz, 2H).

High throughput electrochemical testing

Prior to the electrolysis, the electrolyte (0.1 M $KHCO_3$, ≥99.95% trace metals basis, Sigma Aldrich) with or without additives was purged with CO_2 (99.999%, Airgas) for at least 30 min. A bipolar membrane (BPM, Fumasep® FBM single film, Fumatech) was used to separate the working and counter electrodes. Platinum wire (99.9%, Sigma Aldrich) was used as the counter electrode. Electrolysis was carried out with a Gamry Reference 600TM potentiostat. All electrochemical data were collected using a Ag/AgCl reference electrode (LF2, Innovative Instruments) and converted to a reversible hydrogen electrode (RHE) scale using the measured solution pH of 6.8. All cells and all solution handling lines were purged with fresh electrolyte and CO_2 between electrolysis to avoid cross-contamination. The surface area of the counter electrodes were about 0.25 cm^2, while the working electrode surface areas were 0.32 cm^2. The "fast" flow rate of electrolyte was 150 µL/s throughout the tests, and" slow" was 20 µL/s. The electrode was heated using a Peltier element (Laird thermal) with a heat sink (by Digi Key) below it, which is controlled by TEC-1161-4A-VIN1-SCREW Meerstetter TEC controller. Prior to each refill/experiment, the surface of electrodes was preheated to the desired temperature. The electrolyte was not refilled into the cell until the software (TEC service software) indicated the temperature was stabilized.

Analytical and Electro-chemistry (HT-ANEC) is an analytical electrochemistry system previously published by our group to efficiently detect a wide range of CO_2R products.[2] At the end of each (photo)electrolysis, gaseous and liquid products were sampled by the robotic sample handling system (RSHS) and analyzed by GC (Thermo Scientific™ TRACE™ 1300) and HPLC (Thermo Scientific UltiMate 3000). Detailed product detection (method) can be found in previous publications.[3,4] For any CO_2 reduction experiments with additives involved, the additives were pre-deposited at room temperature at the potential -1.2 V vs RHE for 7.5 min in 0.1 M $KHCO_3$ with 10 mM diphenyliodonium triflate and 10 mM of additive was also present in the 0.1 M $KHCO_3$ during electrocatalysis.

COMSOL Modeling Information

The governing equations for laminar flow and heat transfer in fluids were solved in COMSOL Multiphysics 6.1, coupled by the nonisothermal flow interface. Buoyant effects were taken into account. The default solver configurations were used with a relative tolerance of 0.001. The modeling domain was discretized using an auto-generated extremely fine tetrahedral mesh with 2,020,386 elements. Inlet velocity boundary conditions were set to fully developed flow and matched to experimental flow rates. The outlet was set to 0 Pa to constrain fluid pressure. Inlet flow was set to room temperature and the electrode surface was set to experimental temperatures.

Parameter estimation for CO_2 reduction

We will follow the procedure of Limaye et al. for parameter estimation.[5] Specifically, using Bayesian inference, we will generate distributions for parameters within a model given the raw data. The model we use is the following model:

$$1/i \ = 1/i_{lim} + 1/i_{kinetic}$$

This model was used by Limaye et al. to represent mass transport limitations at high overpotentials, where i is the total current, i_{lim} is the transport-limited current, and $i_{kinetic}$ is the true kinetic current. For the kinetic model, we will use:

$$i_{kinetic} = Ae^{-E_a/RT + \alpha FE/RT}$$

$$ln(i_{kinetic}) = ln(A) - E_a/RT + \alpha FE/RT$$

This is the Butler-Volmer model with temperature dependence included. Here, E_a is the activation energy for the reaction, R is the ideal gas constant, T is the temperature, α is the transfer coefficient (the inverse of the Tafel slope, m_T), A is the prefactor of the Arrhenius rate constant expression, F is Faraday's constant, and E is the applied potential versus RHE. Note that by construction, the potential is positive and higher potentials lead to higher currents. A few important notes about this expression. (1) the prefactor, A, represents the product of multiple prefactors, rate constants, and equilibrium constants in the case of a multi-step reaction where the rate-determining step is not the first electron transfer. (2) Similarly, the activation energy, E_a , is not the activation energy of rate-determining step, necessary, but instead represents a sum of all the Arrhenius expressions that go into the rate constant pre-factor, including the Gibbs free energies for equilibrium reactions before the rate-determining step. (3) the potential is shown as relative to RHE, but in practice could be shown as relative to any reference; the only thing that changes will be the exact value of the activation energy, modified by $\alpha E_{reference}$. Thus, the activation energy reported is actually a function of reference chosen for the system. In the case where the rate-determining step is the first elementary reaction in a multi-step reaction, then the activation energy and the prefactor correspond to the expected values for an elementary step.

We can now modify the above expression with some math to get:

$$ln(i) = ln(i_{lim}) + ln(i_{kinetic}) - ln(i_{lim} + i_{kinetic})$$

$$ln(i) = ln(i_{lim}) + ln(A) - E_a/RT + \alpha FE/RT - ln(i_{lim} + Ae^{-E_a/RT + \alpha FE/RT})$$

Numerically, fitting this can cause problems, so instead we will fit a variation:

$$ln(i_{kinetic}) - ln(i_0) = ln(A) - E_a/RT + \alpha FE/RT - [ln(A) - E_a/RT_0 + \alpha FE_0/RT_0]$$

Essentially, we are now fitting a model where we have normalized the current by the "true" kinetic model. Of course, we do not have the "true" kinetic model, so we instead will use reference potential, temperature, and current from the experimental dataset. Our kinetic model becomes:

$$ln(i_{kinetic}) - ln(b) = -E_a/RT + \alpha FE/RT - [-E_a/RT_{0,exp} + \alpha FE_{0,exp}/RT_{0,exp}]$$

Here, b represents the *actual* kinetic current at the experimentally given reference potential and temperature indicated. Doing this removes some of the arbitrariness from the potential reference and gives physical meaning to the free parameter b instead of a pre-exponential factor A. Empirically, we found that this led to faster and more reproducible fitting with Monte Carlo sampling of the distributions (full parameter fits and distributions Figure S10).

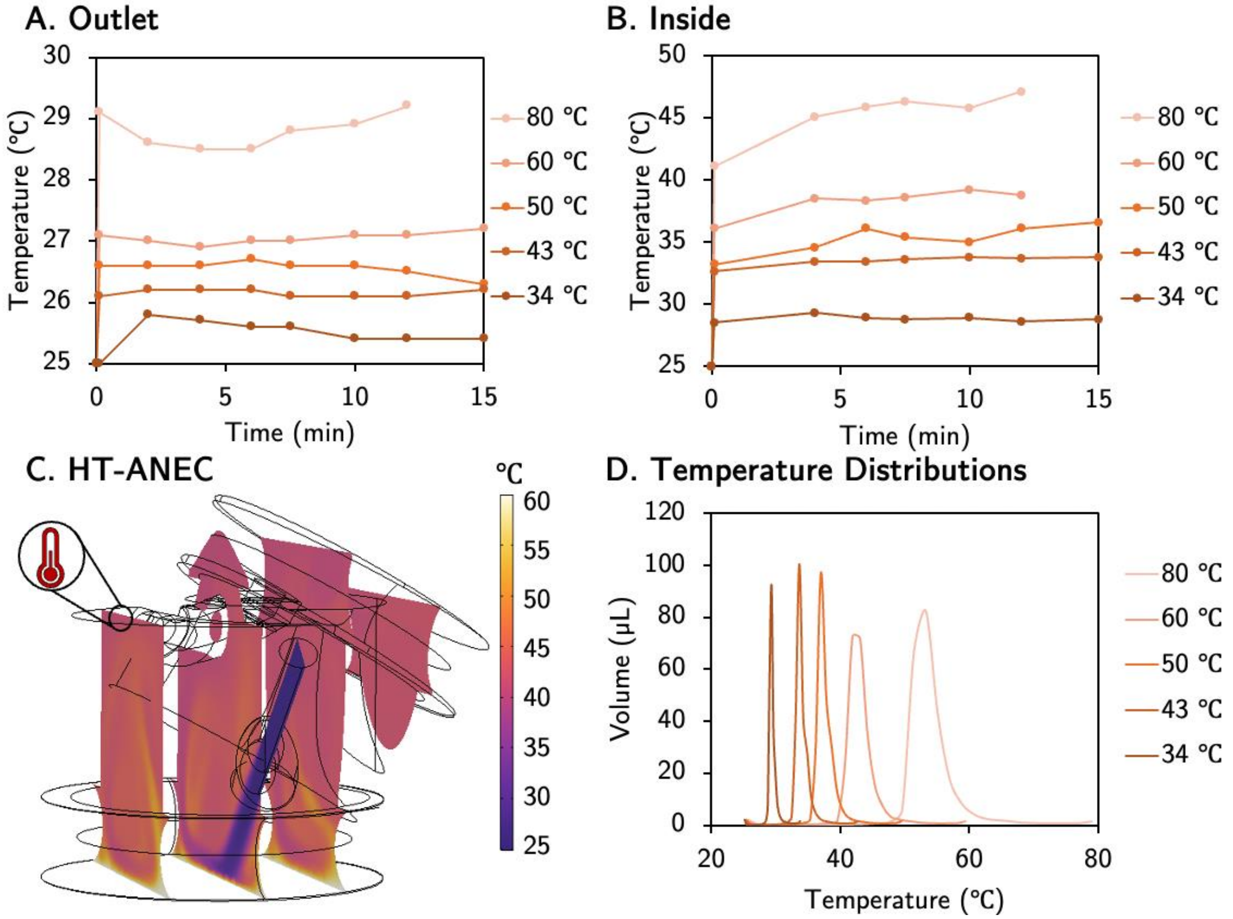

Figure S1: Electrolyte temperature profiles A) 3 cm past the outlet of the cell given variable surface heating temperatures and B) inside the cell. C) Cross-sectional COMSOL image of HT-ANEC with a flow rate of 150 µL/s and SH=60 °C shows decent agreement with experimentally derived values, as shown in table S1. In the inset is the location of the thermocouple for the internal temperature measurements. D) A histogram describing the volume of electrolyte exhibiting a temperature gradient within the cell.

Surface T	Expt outlet T	Expt internal T	<COMSOL> internal	COMSOL std dev	Δ
25	25	25.0	25.0	0.0	0.0
34	25.4	28.6	29.5	0.9	0.9
43	26.2	33.7	34.1	1.9	0.4
50	26.4	36.2	37.8	2.6	1.6
60	27.2	38.8	43.2	3.6	4.4
80	29.1	46.3	53.9	5.6	7.6

Table S1: Comparison of experimental outlet and internal temperatures with COMSOL values. Δ corresponds to the difference between experimental and simulation temperatures. We expect the higher average temperature observed at elevated temperatures in our simulation is due to insufficient equilibration time, as indicated by the positive slopes for 60 and 80 °C. Furthermore, the thermocouple is placed at the top of the cathodic chamber and will therefore read cooler temperatures than the average, which considers the electrolyte closest to the heated electrode.

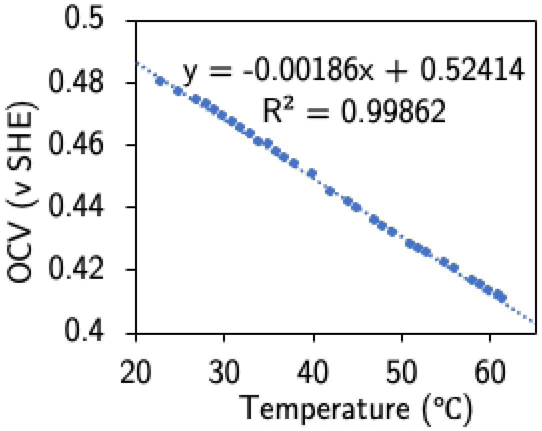

Figure S2: Calibration of OCV versus bulk temperature. Measurements in 0.5 M KCl with 5 mM $K_3Fe(CN)_6$ and 5 mM $K_4Fe(CN)_6$ using a platinum rotating disk electrode, with a platinum wire counter electrode, and a SCE reference electrode.

Set T	25	34	43	50	60
OCV_{BH}	0.478	0.461	0.444	0.431	0.413
$\langle OCV_{fast} \rangle$	0.482 ± 0.000	0.465 ± 0.006	0.452 ± 0.005	0.444 ± 0.007	0.420 ± 0.000
$\langle T_{fast} \rangle$	22.5 ± 0.2	31.9 ± 3.2	39.0 ± 2.6	43.2 ± 3.6	55.9 ± 0.0
$\langle OCV_{slow} \rangle$	0.483 ± 0.001	0.464 ± 0.006	0.450 ± 0.005	0.440 ± 0.005	0.413 ± 0.003
$\langle T_{slow} \rangle$	22.1 ± 0.5	32.3 ± 3.0	39.8 ± 2.6	45.5 ± 2.5	59.6 ± 1.4

Table S2: Open Circuit Voltage (OCV) measurements in 0.5 M KCl with 5 mM $K_3Fe(CN)_6$ and 5 mM $K_4Fe(CN)_6$ shown in Figure 2. BH corresponds to projected values based on a linear regression of data points shown in Figure S2. All slow/fast values are the average of two data points.

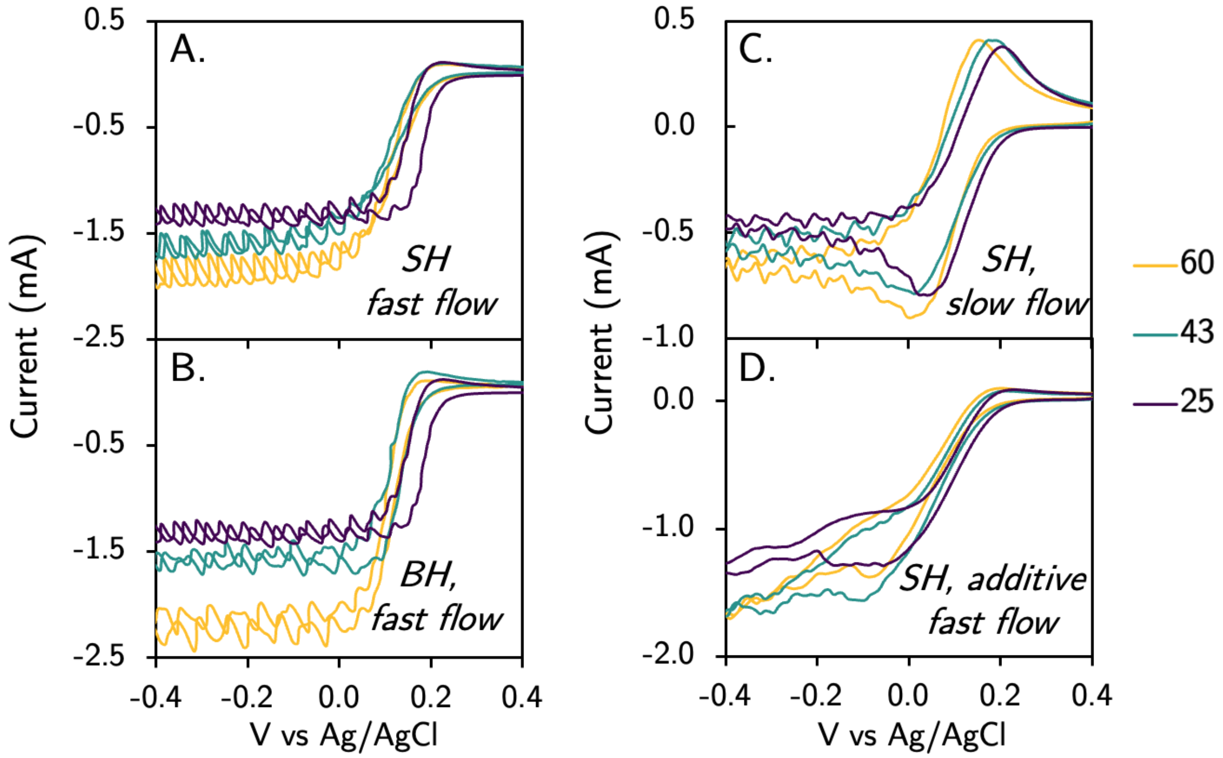

Figure S3: Zoomed out cyclic voltammograms (CVs) of A) surface heating with fast electrolyte recirculation, B) bulk heating with fast electrolyte recirculation, C) surface heating with slow electrolyte recirculation, and D) surface heating with fast electrolyte recirculation in the presence of a molecular film (deposited in the same way as otherwise referred to in the text). Cyclic voltammograms were performed with 10 mM $K_3Fe(CN)_6$ in CO_2-sparged 0.1 M $KHCO_3$ scanning at 100 mV/s from 0.5 V to -1 V vs Ag/AgCl with a gold working electrode, Pt counter electrode, and leakless Ag/AgCl reference electrode. CVs were performed to establish a mass transport limited regime for subsequent chronoamperometry experiments. The small fluctuations in current observed are associated with the periodicity of the barrels in the peristaltic pump pushing the electrolyte through the system.

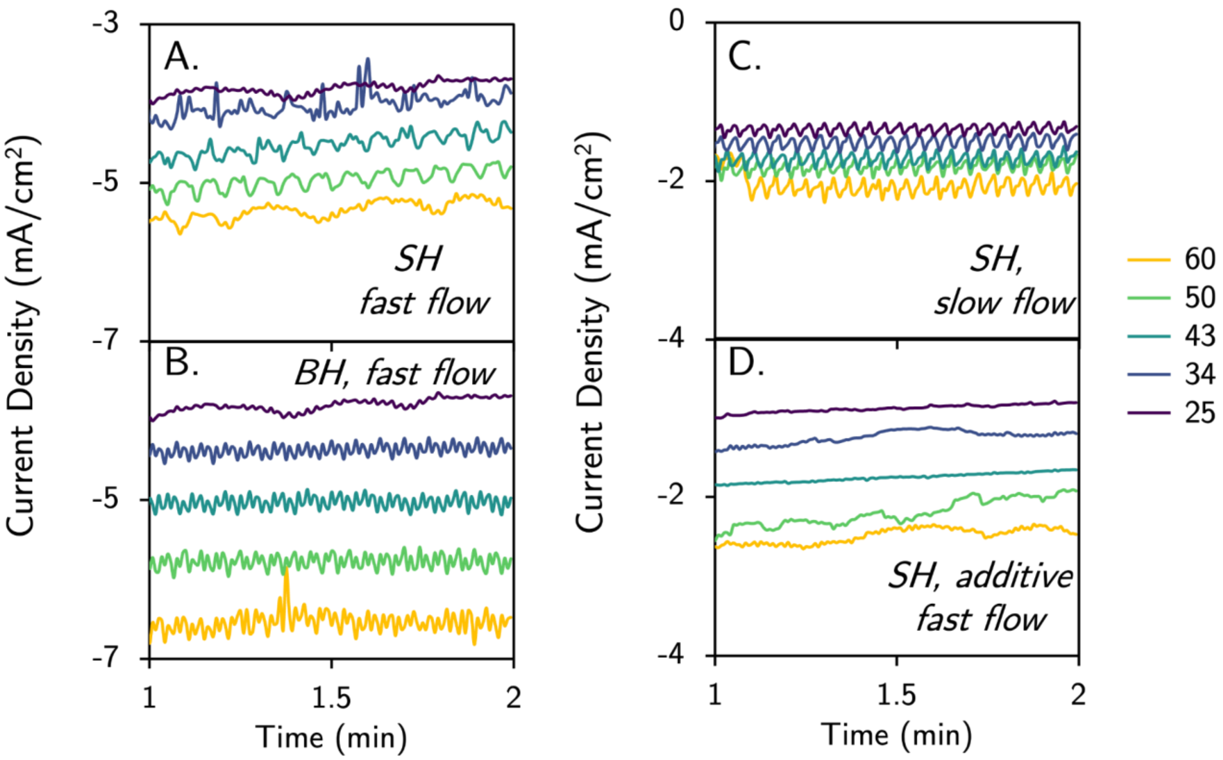

Figure S4: Chronoamperometry (CA) at -0.8 V vs Ag/AgCl for 2 minutes of A) surface heating with fast electrolyte recirculation, B) bulk heating with fast electrolyte recirculation, C) surface heating with slow electrolyte recirculation, and D) surface heating with fast electrolyte recirculation in the presence of a molecular film (deposited in the same way as otherwise referred to in the text). CAs were performed with 10 mM $K_3Fe(CN)_6$ in CO_2-sparged 0.1 M $KHCO_3$ with a gold working electrode, Pt counter electrode, and leakless Ag/AgCl reference electrode. The average current across the second minute was used in subsequent boundary layer calculations. The significant decrease in activity with organic films may be due to the inhibition of the transfer of ferricyanide through the film. The small fluctuations in current observed are associated with the periodicity of the barrels in the peristaltic pump pushing the electrolyte through the system.

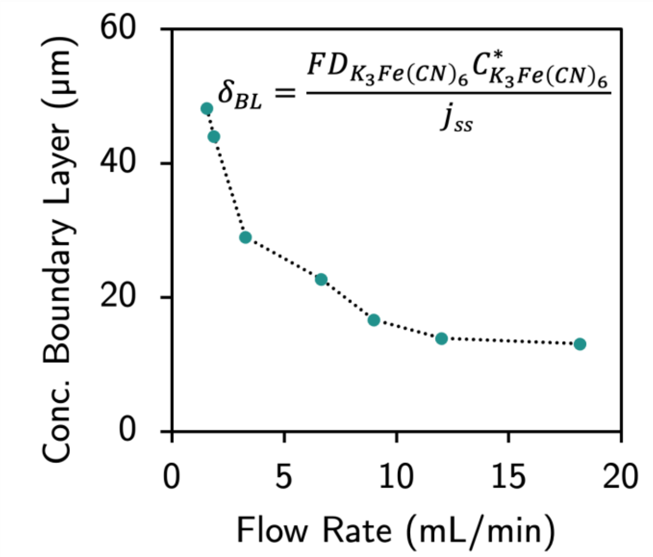

Figure S5: Concentration boundary layer versus electrolyte recirculation flow rate. These experiments were obtained according to section SI-4 from Clark et al.[6] Briefly, a CV is initially performed to determine the mass transport limited regime, and then a potential is chosen for subsequent constant potential experiments to determine the boundary layer at different recirculation rates. The boundary layer was calculated using the inset equation, wherein $D_{K3Fe(CN)6}$ is the diffusivity of the ferricyanide ion at 25 °C (0.72×10^{-5} cm^2 s^{-1}), $C^*[Fe(CN)_6{}^{3-}]$ is the concentration of ferricyanide ion in the bulk of the electrolyte (10 mM), and j_{SS} is the steady-state current density.

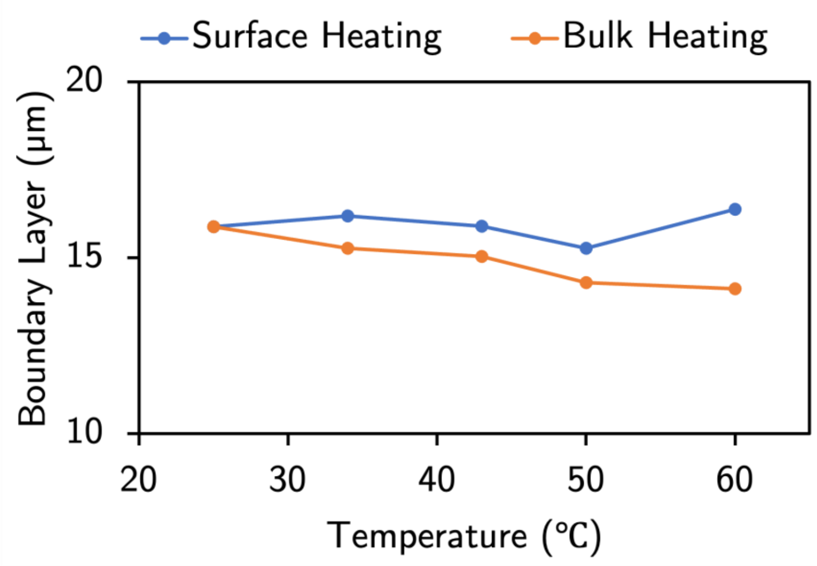

Figure S6: Calculated concentration boundary layer thickness at a 150 μL/s flow rate using surface temperatures from Table S2 and temperature-adjusted diffusion coefficients.[7]

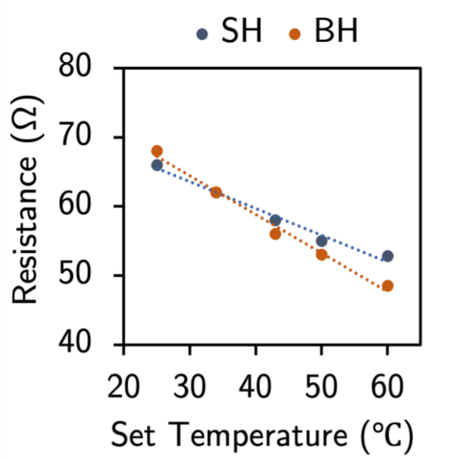

Figure S7: Temperature versus resistance plots of surface heated (SH) versus bulk heated (BH) electrolyte using 0.1 M $KHCO_3$.

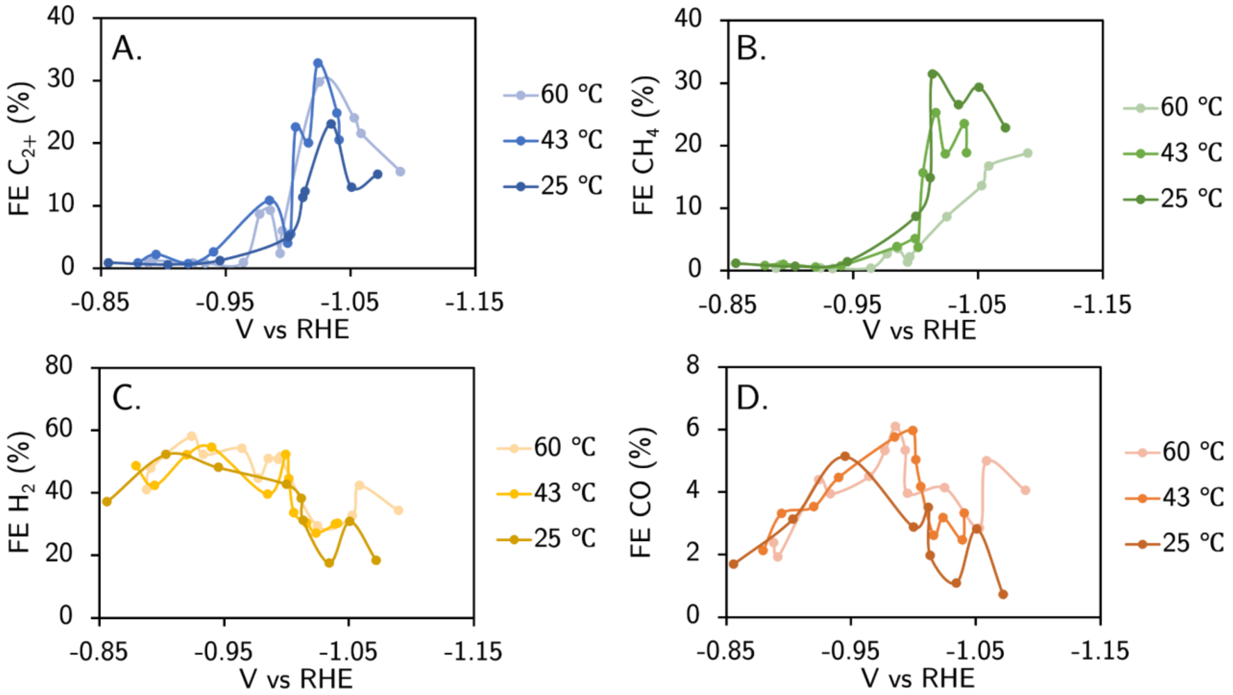

Figure S8: Plots of Faradaic efficiencies for polycrystalline Cu CO_2R at variable temperatures in 0.1 M $KHCO_3$. Each data point corresponds to an individual experiment.

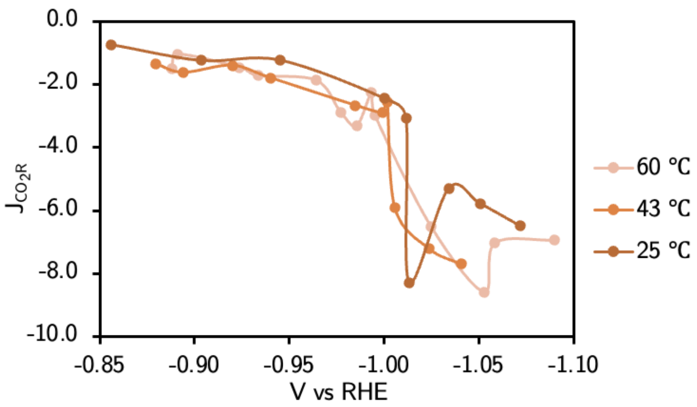

Figure S9: Partial current density towards electrochemical CO_2 reduction at variable temperatures in the absence of molecular coating in 0.1 M $KHCO_3$ on polycrystalline Cu. Each data point corresponds to an individual experiment.

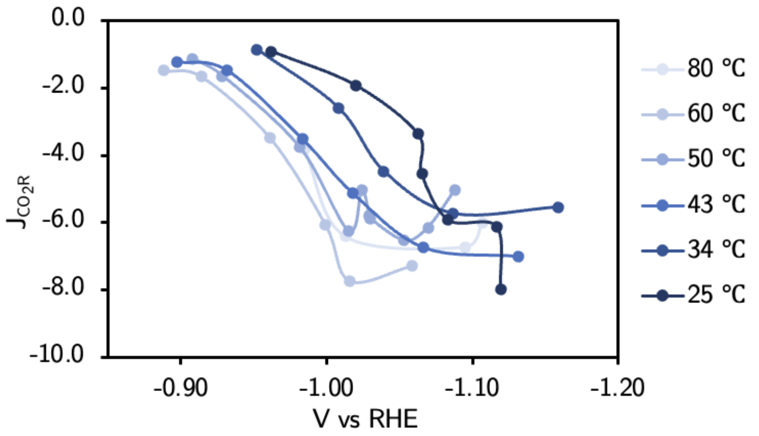

Figure S10: Partial current density towards electrochemical CO_2 reduction at variable temperatures in the absence of molecular coating in 0.1 M $KHCO_3$ on polycrystalline Cu with an organic coating derived from diphenyliodonium triflate. Each data point corresponds to an individual experiment.

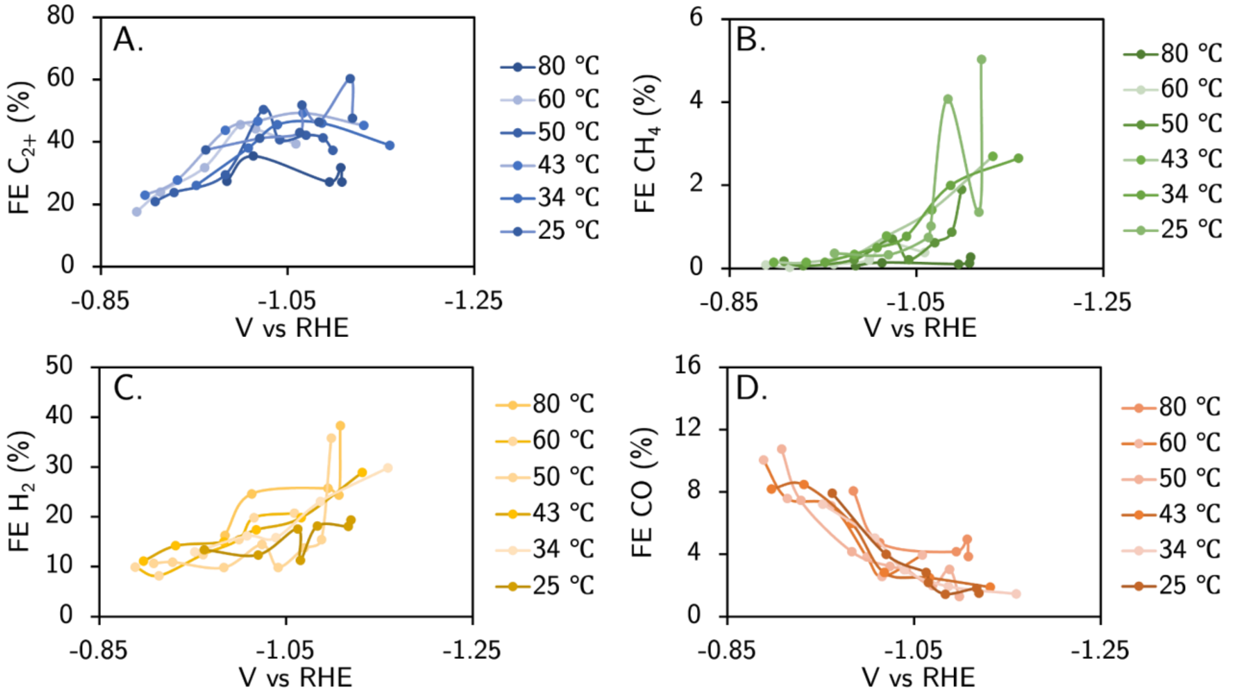

Figure S11: Plots of Faradaic efficiencies for organic-modified polycrystalline Cu CO_2R at variable temperatures in 0.1 M $KHCO_3$. Each data point corresponds to an individual experiment.

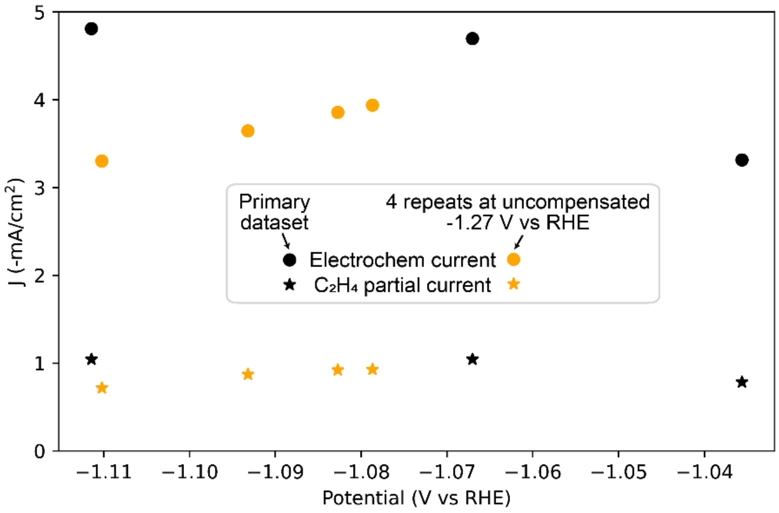

Figure S12: The reproducibility of C_2H_4 partial current density with 60 °C surface heating and an uncompensated applied potential of -1.27 V vs RHE. Due to the cell resistance and variation of the total current density (3.7 ± 0.3 mA cm^{-2}), the resistance-compensated potential is slightly different for the 4 experiments. Still, the C_2H_4 partial current density appears quite reproducible mean and standard deviation of 0.86 ± 0.10 mA cm^{-2}. To place these 4 repeat measurements in the context of the primary dataset, the 3 nearby potentials with 60 °C surface heating are also shown in the figure. The C_2H_4 partial current density for this potential range is consistent across the data. The 4 repeat experiments were performed with a copy of the original electrochemical cell and a different lot of copper foil. We confirmed that the reference-working resistance used for resistance compensation remains unchanged from the cell used for the primary dataset. We anticipate that the systematically lower electrochemical current results from subtle differences in the copper foil, which apparently are not very impactful for C_2H_4 partial current density. The 4 repeat measurements show adequate reproducibility of the C_2H_4 partial current density for a given catalyst and condition, validating our use of Bayesian modelling of the ensemble of electrochemical conditions to quantify uncertainty in the temperature-dependent Tafel equation.

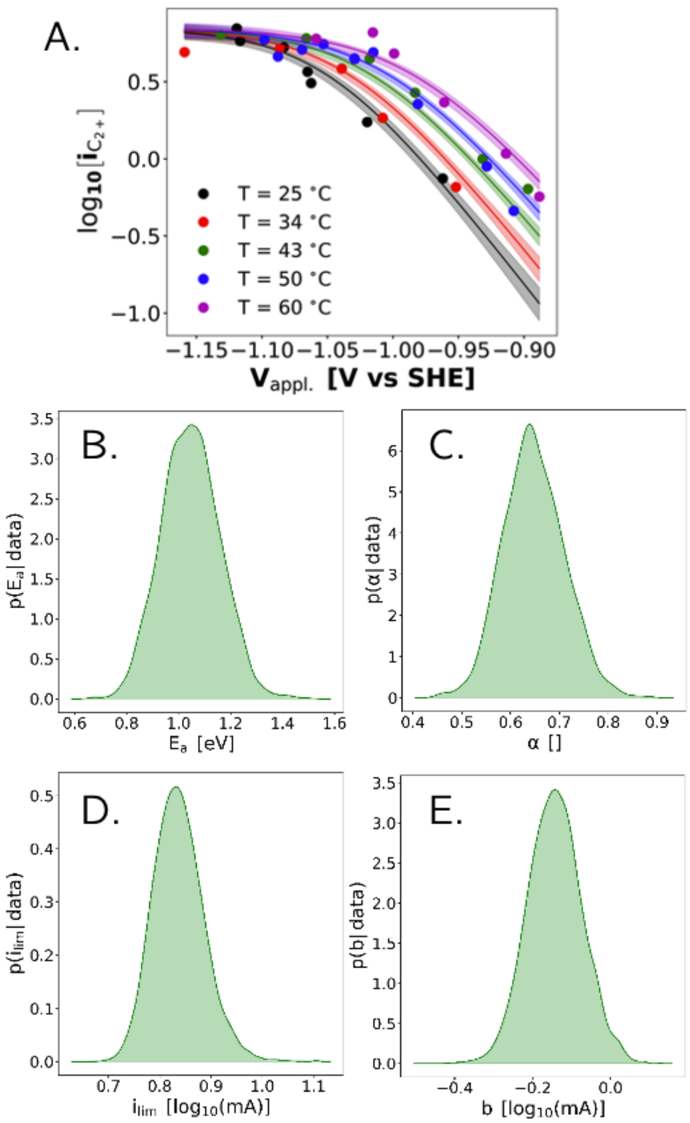

Figure S13: Fitting of experimental CO_2R data using Tafel kinetics (Equation 2 in the main text and full model on SI page 3). (A) The data points from experiments are shown along with the fitted Tafel model, where the solid lines correspond to the maximum likelihood Tafel parameters. The shaded regions illustrate the one-sigma confidence interval of the fit given the posterior probability distribution functions of all parameters from the Bayesian model. (B-E) The posterior probability distribution functions are shown for the activation energy (B), the transfer coefficient (C), the limiting current (D), and the pre-factor (E). All variables and fit equations are defined above in full model discussion.

Table S3: Potentials, currents, temperatures, and Faradaic efficiencies of electrolyses reported herein.

T (°C)	[Additive] (mM)	V vs RHE	J total (mA/cm²)	H_2	CH_4	CO	C_2H_4	EtOH	CHOOH	C_{2+} Liq.	Total FE
25	-	-0.86	-2.23	37.0	1.2	1.7	0.9	0.0	0.0	0.0	40.8
25	-	-0.90	-2.36	52.2	0.7	3.1	0.6	0.0	0.0	0.0	56.6
25	-	-0.95	-2.77	48.0	1.5	5.1	1.2	0.0	0.0	0.0	55.9
25	-	-1.00	-5.06	42.6	8.7	2.9	3.4	0.0	0.0	1.7	59.2
25	-	-1.01	-7.00	38.2	14.9	3.5	5.4	1.5	0.0	4.5	67.9
25	-	-1.01	-13.25	31.1	31.4	2.0	7.0	1.5	0.7	3.8	77.4
25	-	-1.03	-8.39	17.4	26.6	1.1	10.7	4.7	0.8	7.7	68.9
25	-	-1.05	-10.10	30.7	29.4	2.8	6.5	1.6	0.5	4.8	76.3
25	-	-1.07	-11.56	18.3	22.9	0.7	6.0	3.7	0.5	5.3	57.4
25	10	-0.96	-1.99	13.4	0.4	7.9	16.8	20.7	0.0	0.0	59.2
25	10	-1.02	-4.21	12.4	0.3	4.0	21.4	18.2	0.0	1.6	57.9
25	10	-1.06	-7.22	17.6	0.7	2.8	31.6	10.9	0.0	0.5	64.2
25	10	-1.07	-7.08	11.3	1.0	2.2	32.4	15.8	9.2	3.7	75.5
25	10	-1.08	-11.39	18.1	4.1	1.4	25.8	19.2	0.0	1.4	70.1
25	10	-1.12	-9.63	18.1	1.4	1.8	31.7	24.8	0.0	3.7	81.6
25	10	-1.12	-14.76	19.3	5.0	1.5	28.5	17.8	0.0	1.3	73.5
34	10	-0.95	-2.52	13.0	0.1	7.2	16.0	2.8	7.9	0.0	47.1
34	10	-1.01	-4.84	16.2	0.5	5.0	21.5	7.2	14.8	4.4	69.6
34	10	-1.04	-8.46	15.8	0.8	3.0	25.4	13.4	6.6	3.7	68.7
34	10	-1.09	-11.22	23.1	2.0	1.9	27.5	15.3	3.0	1.3	74.1
34	10	-1.16	-12.66	29.8	2.7	1.5	23.2	13.0	2.1	1.3	73.5
43	-	-0.88	-4.18	48.5	0.9	2.1	0.8	0.0	28.2	0.0	80.5
43	-	-0.89	-3.31	42.2	1.1	3.3	1.4	0.0	42.3	0.8	91.1
43	-	-0.92	-4.74	52.0	0.6	3.5	0.7	0.0	24.5	0.0	81.4
43	-	-0.94	-3.55	54.5	0.8	4.5	1.5	0.0	42.4	1.1	104.7
43	-	-0.98	-5.34	39.5	3.8	5.8	4.2	0.7	29.4	5.9	89.3
43	-	-1.00	-5.96	52.1	5.1	6.0	4.0	0.0	33.4	0.0	100.7
43	-	-1.00	-5.81	44.4	3.7	5.0	4.2	0.0	29.5	1.2	88.2
43	-	-1.01	-11.50	33.6	15.7	4.2	10.4	2.2	9.0	10.0	85.0
43	-	-1.02	-16.81	30.3	25.3	2.6	10.9	2.0	9.8	7.1	88.0
43	-	-1.02	-10.44	27.0	18.7	3.2	16.0	4.7	14.3	12.2	96.0
43	-	-1.04	-15.46	29.9	23.5	2.5	12.9	3.4	21.1	8.6	101.9
43	-	-1.04	-12.40	30.1	18.9	3.3	9.5	2.9	19.4	8.2	92.3
43	10	-0.90	-2.78	11.2	0.1	8.2	9.3	3.2	20.8	2.2	55.1
43	10	-0.93	-3.59	14.2	0.1	8.5	13.8	4.1	13.1	1.4	55.3
43	10	-0.98	-6.14	15.0	0.3	6.0	24.0	7.3	13.2	6.5	72.2
43	10	-1.02	-9.57	17.4	0.8	2.8	26.8	13.8	6.1	3.3	71.0
43	10	-1.07	-12.29	19.9	1.4	2.5	27.4	15.9	4.1	3.5	74.7
43	10	-1.13	-14.12	28.9	2.7	1.9	27.1	14.9	1.6	1.4	78.5
50	10	-0.91	-2.21	10.7	0.2	10.8	13.1	5.3	19.4	2.5	61.8
50	10	-0.93	-3.77	10.9	0.1	7.5	13.1	5.8	12.2	4.9	54.4
50	10	-0.98	-7.57	19.1	0.1	7.5	17.6	7.8	11.9	4.5	68.7
50	10	-1.02	-11.79	24.0	0.2	4.4	21.5	12.9	6.7	7.3	77.1
50	10	-1.02	-9.25	14.4	0.7	3.2	26.6	16.9	6.7	7.0	68.8
50	10	-1.03	-10.89	21.3	0.2	4.9	21.0	12.6	7.3	7.3	74.4
50	10	-1.03	-10.86	22.3	0.1	4.9	21.8	13.3	7.7	6.2	76.4
50	10	-1.05	-12.54	21.1	0.2	4.0	25.4	13.5	3.6	5.4	73.1

50	10	-1.07	-12.12	13.8	0.6	2.0	20.5	16.9	5.9	4.8	64.5
50	10	-1.09	-11.16	15.5	0.9	3.0	20.4	14.3	7.4	6.7	60.7
50	10	-1.10	-15.86	35.8	1.9	1.3	20.0	14.2	1.9	3.1	78.2
60	-	-0.89	-3.95	40.9	0.4	2.4	0.9	0.0	0.0	0.0	44.6
60	-	-0.89	-3.73	47.8	1.0	1.9	1.2	0.0	0.0	0.0	51.9
60	-	-0.92	-4.85	58.0	0.5	4.4	0.9	0.0	0.0	0.0	63.8
60	-	-0.93	-4.24	52.2	0.4	3.9	0.8	0.0	0.0	0.0	57.3
60	-	-0.96	-5.46	54.1	0.4	4.5	0.9	0.0	0.0	0.0	59.9
60	-	-0.98	-6.23	44.6	2.7	5.3	3.9	1.2	0.3	3.6	61.6
60	-	-0.99	-7.28	50.8	3.6	6.1	4.1	0.7	0.6	4.5	70.4
60	-	-0.99	-6.78	50.7	1.4	5.3	2.4	0.0	0.0	0.0	59.8
60	-	-1.00	-6.67	51.4	2.2	4.0	2.9	0.0	0.0	3.1	63.6
60	-	-1.02	-11.14	29.3	8.6	4.1	13.4	3.9	1.6	12.6	73.4
60	-	-1.05	-15.73	32.7	13.6	2.8	11.0	3.0	1.3	10.0	74.5
60	-	-1.06	-12.18	42.2	16.7	5.0	12.3	1.7	0.7	7.6	86.2
60	-	-1.09	-13.38	34.3	18.8	4.0	9.4	1.6	0.2	4.5	72.8
60	10	-0.89	-3.24	10.0	0.1	10.0	9.3	4.2	18.1	4.2	55.8
60	10	-0.91	-4.53	8.2	0.0	7.6	9.9	5.8	5.0	8.2	44.7
60	10	-0.96	-7.31	12.4	0.1	7.1	20.2	6.4	8.6	5.3	60.1
60	10	-1.00	-10.56	15.4	0.2	3.8	23.6	15.1	8.0	6.8	73.0
60	10	-1.02	-14.96	19.8	0.6	2.6	22.2	16.0	4.3	6.0	71.6
60	10	-1.06	-15.32	20.7	0.4	4.0	21.7	12.5	3.9	5.1	68.3
80	10	-0.98	-8.57	16.3	0.1	8.1	13.9	5.9	9.0	7.6	60.8
80	10	-1.01	-13.88	24.6	0.1	4.7	19.6	10.4	5.8	5.6	70.8
80	10	-1.09	-15.24	25.7	0.1	4.2	13.3	8.5	12.7	5.4	69.8
80	10	-1.11	-14.36	24.3	0.1	5.0	16.5	9.0	5.1	6.2	66.2
80	10	-1.11	-17.98	38.3	0.3	3.9	15.3	8.6	4.7	3.3	74.3

References

(1) Bielawski, M.; Olofsson, B. High-Yielding One-Pot Synthesis of Diaryliodonium Triflates from Arenes and Iodine or Aryl Iodides. *Chem. Commun.* **2007**, No. 24, 2521–2523. https://doi.org/10.1039/B701864A.

(2) Jones, R. J. R.; Wang, Y.; Lai, Y.; Shinde, A.; Gregoire, J. M. Reactor Design and Integration with Product Detection to Accelerate Screening of Electrocatalysts for Carbon Dioxide Reduction. *Rev. Sci. Instrum.* **2018**, *89* (12), 124102. https://doi.org/10.1063/1.5049704.

(3) Lai, Y.; Jones, R. J. R.; Wang, Y.; Zhou, L.; Richter, M. H.; Gregoire, J. The Sensitivity of Cu for Electrochemical Carbon Dioxide Reduction to Hydrocarbons as Revealed by High Throughput Experiments. *J. Mater. Chem. A* **2019**, *7* (47), 26785–26790. https://doi.org/10.1039/C9TA10111J.

(4) Lai, Y.; Watkins, N. B.; Muzzillo, C.; Richter, M.; Kan, K.; Zhou, L.; Haber, J. A.; Zakutayev, A.; Peters, J. C.; Agapie, T.; Gregoire, J. M. Molecular Coatings Improve the Selectivity and Durability of CO2 Reduction Chalcogenide Photocathodes. *ACS Energy Lett.* **2022**, *7* (3), 1195–1201. https://doi.org/10.1021/acsenergylett.1c02762.

(5) Limaye, A. M.; Zeng, J. S.; Willard, A. P.; Manthiram, K. Bayesian Data Analysis Reveals No Preference for Cardinal Tafel Slopes in CO 2 Reduction Electrocatalysis. *Nat. Commun.* **2021**, *12* (1), 703. https://doi.org/10.1038/s41467-021-20924-y.

(6) Clark, E. L.; Resasco, J.; Landers, A.; Lin, J.; Chung, L.-T.; Walton, A.; Hahn, C.; Jaramillo, T. F.; Bell, A. T. Standards and Protocols for Data Acquisition and Reporting for Studies of the Electrochemical Reduction of Carbon Dioxide. *ACS Catal.* **2018**, *8* (7), 6560–6570. https://doi.org/10.1021/acscatal.8b01340.

(7) Arvía, A. J.; Marchiano, S. L.; Podestá, J. J. The Diffusion of Ferrocyanide and Ferricyanide Ions in Aqueous Solutions of Potassium Hydroxide. *Electrochimica Acta* **1967**, *12* (3), 259–266. https://doi.org/10.1016/0013-4686(67)80004-5.
